# Progress in State Medicine

**Published:** 1901-02-16

**Authors:** 


### Progress in State Medicine.

### HOUSING OF THE WORKING CLASSES.

"The[1] greatest hindrance to the progress of preventive medicine which we have to overcome is the overcrowded and insanitary condition of the dwellings of the working classes." This statement was quoted by Tew[2] whilst speaking on the provision of cottages in rural districts for the working classes and its bearing upon the public health. Combat is not directly with the microbe, but with the surroundings in which it thrives. To determine what is a rural district he favoured some such division as the following: (1) Metropolis proper; (2) suburban districts where the question of accommodation was somewhat less acute than in the centre of London; (3) extra-suburban districts, *i.e.* just beyond the immediate suburbs, but which were fast being absorbed into the suburbs of the spreading metropolis; (4) agricultural districts where the inhabitants worked on the land; (5) cities and large provincial towns where special conditions of situation, industries, &c., made the question of provision of further dwelling accommodation purely local; (6) suburbs of cities and large provincial towns which must be dealt with on their own merits. Of these 3, 4, and 6 would come under the heading of rural districts. The working people resolve themselves into three classes: (1) In good work, wages 25s. to 30s. a week, but whose greatest difficulty is in procuring decent dwellings, rents being from 6s. 6d. to 8s. 6d. a week. This led to the inevitable lodgers, and consequent overcrowding. (2) Ordinary agricultural labourers, wages 12s. to 22s. On large estates this class is often well housed, rent 1s. to 2s. 6d. per week, with ga[illegible] and supply of milk. In others the cottages might be cheap, under 2s., but were small, damp, and ill-drained. The remainder lived in their own cottages, which are generally mortgaged, and rarely in a satisfactory condition. (3) Those having no regular occupation, and having an alternating existence between a slum cottage, the workhouse, the gaol, and the road. The children of these were active agents in the spread of infectious and parasitic diseases in our elementary schools. The foremost evil was the lowering of the individual stamina of those living under bad conditions; next the two diseases—measles and whooping cough—which in 1899 accounted for 19,000 deaths, mostly among the children of the working classes. Obstacles to the provision of adequate cottages were many and great, and although increased powers had been available for ten years, only two rural district councils in England had reached the state of actual buildings. The annual re-election of sanitary inspectors and medical officers of health was often jeopardised by local interests. Fresh houses should be provided before closing insanitary ones. Where land was not big-priced rural councils could build cottages just to pay their way, but even should there be a loss on them such loss must be looked on as an insurance premium on the health of the place. Groves[3] is of opinion that the landlords receiving such small returns as rent could not afford repairs, and so forced the labourers from the condemned houses into the nearest towns. Security of tenure for all sanitary officials was the only safeguard, and that could be obtained only from the Local Government Board. Welton[4] draws attention to the fact that at the time of the first census 36 per cent. of the people lived in towns of 1,000 inhabitants and upwards, but in 1891 64 per cent. of the people lived in towns exceeding 4,000 in population. The ordinary density of population in rural districts was in 1801 about 100 persons per square mile; in 1891 it averaged 130 persons. Since 1851 there has been an actual decline in rural population.

Pattin[5] remarks that by workmen's dwellings he means homes suitable for poor men rather than artisans, or skilled artisans. The need for action is emphasised by the fact that, in Manchester alone, out of 11,000 would-be recruits, 90 per cent. were excluded from the regular army on the ground of physical unfitness. Inefficiently-equipped and managed common lodging-houses damaged the health of the community. The credit of the municipality is greater than that of the individual who is trading for a profit, and therefore it is easier for it to carry on common lodging-houses and provide poor men's cottages more cheaply than the private individual. The advantages from this would be an improved generation through healthier surroundings, and more air space; also the importation and propagation of various communicable diseases would be more easily checked. The arguments brought against municipal management are that it would interfere with private enterprise, and would add to already large municipal debts. The municipality should provide common lodging-houses with the minimum amount of comfort, and leave it to private enterprise to attract custom by providing further comfort. He apprehended that the housing of the really poor could not be expected to prove a directly profitable undertaking to any municipality; the profit would be indirect, but enduring.

Tew[6] mentions that the Liverpool cheap dwellings had not been a financial success, and adds that the municipal dwellings erected in London and other large centres cost an amount requiring a rent of 2s. to 3s. a room to pay their way. The three- or four-room dwellings erected by the Richmond Corporation, and letting at a rent of 4s. 6d. and 5s. 6d. respectively, were comfortable, healthy, and self-supporting.

Provis,[7] in a letter to the municipal authorities on the subject of the New Housing Act, points out that when any council other than a rural district council has adopted Part III. of that Act (the part relating to municipal lodging-houses), it may for supplying the needs of its district establish or acquire lodging-houses for the working-classes, under that part, outside its district. This was illegal before. Further, that the new Act will enable the council, with the consent of the board, to lease any land acquired by them under, and for the purposes of, Part III. to any lessee, for the purpose and on condition that he will carry the Act into execution by building and maintaining on the land lodging-houses within the meaning of the Act.

Berry[8] says that the most insanitary dwellings exist in the smaller towns and villages. The older the town the worse the evil. Reviewing the Housing of the Working Classes Act (1890) he is of opinion that under Part I. (that referring to unhealthy areas), whilst it is sometimes desirable to condemn such insanitary areas, it is a costly method which the smaller towns seek to avoid. Part II. (which refers to unhealthy dwellings) is more suitable for small towns and villages, for under Part I. dwelling accommodation must be provided for the displaced tenants. Part III. (referring to provision of working-class lodging houses and dwellings) is adoptive, but is not taken advantage of as it might. If local authorities would only enforce the powers already possessed under these Acts, much more progress would be made. He agrees with Sir Charles Cameron[9] that the closing of insanitary houses and clearing unhealthy areas have been of little benefit to the poorest of the poor. The rents of the new dwellings were beyond their means. Fyfe[10] thinks much might be done by the inspectors in educating the poorer classes in things hygienic. The houses may be bad, but so are often the tenants.

In Glasgow six female inspectors were appointed who were authorised to pass orders to poor persons, securing them gratis as much whiting and colouring powder as were needful, the loan of a brush wherewith to brighten and cleanse their homes. It is difficult now in the city of Glasgow to find a really filthy house. Persuasion will effect more than legal proceedings.

[1] Brit. Med. Jour., Aug 5, 1899. [2] Lancet, Aug. 11, 1900. [3] Ibid. [4] Brit. Med. Jour., Dec. 22, 1900. [5] Brit. Med. Jour., Aug. 18, 1900. [6] Ibid. [7] Municipal Journal, Aug. 31, 1900. [8] Brit. Med. Journ., Aug. 18, 1900. [9] Journal of State Medicine, 1898. [10] Sanitary and Municipal Engineering, Aug. 31, 1900.

